# Lung inflammatory pattern and antibiotic treatment in pneumonia

**DOI:** 10.1186/s12931-015-0165-y

**Published:** 2015-02-07

**Authors:** María-José Lorenzo, Inés Moret, Benjamín Sarria, Enrique Cases, Julio Cortijo, Raúl Méndez, Jose Molina, Alejandra Gimeno, Rosario Menéndez

**Affiliations:** Pneumology Department, Instituto de Investigación Sanitaria del Hospital Universitario y Politécnico La Fe (IIS La Fe), Valencia, Spain; Instituto de Investigación Sanitaria del Hospital Universitario y Politécnico La Fe (IIS La Fe), Valencia, Spain; Pharmacology Department, University of Medicine, Universidad de Valencia, Valencia, Spain; Medicine-Doctoral Programme Department, Barcelona Autonomous University, Barcelona, Spain; Research contract IIS La Fe-Fundación Bancaja, Valencia, Spain; Microbiology Department, Hospital Universitario y Politécnico La Fe, Valencia, Spain; CIBER de Enfermedades Respiratorias (CibeRes), Valencia, Spain; Servicio de Neumología, Hospital Universitario y Politécnico La Fe, Avda Bulevar Sur, 46026 Valencia, Spain

**Keywords:** Community acquired pneumonia, Macrolides, Lung inflammation

## Abstract

**Background:**

In community-acquired pneumonia host inflammatory response against the causative microorganism is necessary for infection resolution. However an excessive response can have deleterious effects. In addition to antimicrobial effects, macrolide antibiotics are known to possess immunomodulatory properties.

We aimed to evaluate inflammatory cytokine profiles – both locally (bronchoalveolar lavage) and systemically (blood) – in community-acquired pneumonia admitted patients after at least 72 hours of antibiotic treatment (with and without macrolide containing regimens) and requiring bronchoscopic examination for inadequate response due to infection progression and/or lack of clinical stability.

**Methods:**

A prospective study was performed on 52 admitted patients who developed an inadequate response after 72 hours of antibiotic treatment - non-responders community-acquired pneumonia - (blood and bronchoalveolar lavage), and two control groups: 1) community-acquired pneumonia control (blood) and 2) non-infection control (blood and bronchoalveolar lavage). Cytokine profiles (interleukin (IL)-6, IL-8, IL-10), tumour necrosis factor α and clinical outcomes were assessed.

**Results:**

Non–responders patients treated with macrolide containing regimens showed significantly lower levels of IL-6 and TNF-α in bronchoalveolar lavage fluid and lower IL-8 and IL-10 in blood than those patients treated with non-macrolide regimens. Clinical outcomes showed that patients treated with macrolide regimens required fewer days to reach clinical stability (p < 0.01) and shorter hospitalization periods (p < 0.01).

**Conclusions:**

After 72 hours of antibiotic effect, patients who received macrolide containing regimens exhibited lower inflammatory cytokine levels in pulmonary and systemic compartments along with faster stabilization of infectious parameters.

## Introduction

The majority of hospitalized community-acquired pneumonia (CAP) patients respond favorably to antibiotic treatment but around 10% develop an inadequate response to treatment that leads to a poorer prognosis [1]. Cytokines are important mediators that orchestrate the inflammatory response and hence play an important role in host defense against microorganisms. However, excessive and persistent cytokine production [2] has been associated with a higher number of days needed to reach clinical stability, treatment failure and increased mortality [3,4]. After antibiotic treatment, when the response is adequate, inflammation decreases with a return to homeostasis.

The immunomodulatory effects of macrolides benefit the host due to their capacity to temper the production of inflammatory cytokines [5-7]. Some recent observational studies have reported better outcomes in severe CAP cases [8-12], when antibiotics regimens contained macrolides. Others studies, though, have not reported such improvement [13,14]. We hypothesized that antibiotic influence on cytokine production might have a beneficiary effect upon the resolution of infectious parameters. Although systemic inflammation has been examined previously, research into lung cytokine patterns in CAP with different antibiotics regimens is scarce.

The aims of the present study were to investigate 1) lung and systemic inflammatory cytokine profiles in hospitalized CAP patients after at least 72 hours of antibiotic treatment (with and without macrolide containing regimens) and who required bronchoscopic examination owing to an inadequate response consequent upon infection progression and/or lack of clinical stability; and 2) the impact of macrolide containing regimens treatment on clinical resolution parameters.

## Methods

### Study design

A prospective longitudinal study was conducted of admitted CAP patients requiring bronchoscopic examination due to an inadequate response to antibiotic treatment after at least 72 hours of antibiotic treatment: non-responders CAP (NCAP). The inclusion criteria for the NCAP study group were: 1) clinical deterioration with acute respiratory failure requiring ventilator support and/or septic shock; and/or 2) persistence of a high temperature (≥38°C) and/or clinical symptoms, and/or chest-X ray progression (>50% increase of infiltrates with clinical symptoms) or empyema [3]. Patient enrolment in the study was performed on the day of their bronchoscopic examination when samples of bronchoalveolar (BAL) fluid and blood were obtained.

Two control groups were included: 1) CAP control group (CAP), comprised of patients of a similar age and co-morbid condition who reached clinical stability after 72 hours of antibiotic treatment (temperature <37.2°C, heart rate <100 beats/min, respiratory rate <24 breaths/min, systolic blood pressure >90 mm Hg, and oxygen saturation >90% or arterial oxygen tension >60 mm Hg when patient was not receiving supplemental oxygen) [15,16]; and 2) Non-infection control group, comprised of patients without any infection for whom a bronchoscopy had been scheduled due to peripheral lung nodules or minor haemoptysis.

Exclusion criteria were: prior admission to hospital (1 month), immunosuppressive treatment, HIV infection, or alternative diagnosis.

The study was approved by the Ethical Committee of our hospital (approval number 2005/0141) and patients signed the required informed consent form.

### Data collection

The following data were collected: demographics, co-morbid conditions, laboratory data, chest radiograph, initial pneumonia severity [17], microbiological tests (sputum culture, urinary antigens of *Legionella pneumophila* and *Streptococcus pneumoniae,* blood cultures and serology for *Chlamydophila pneumoniae, Mycoplasma pneumonia, Coxiella burnetii* and *Legionella pneumophila)*, and length of hospital stay (LOS) which was calculated as the number of days from admission until discharge.

Antimicrobial therapy received by patients was recorded and classified as follows: beta-lactams (ceftriaxone/cefotaxime: 1–2 g/12-24 h, 1–2 g/8 h or co-amoxiclavulanate: 1–2 g/8 h) + macrolides (azithromycin: 500 mg/24 h); fluoroquinolone (levofloxacin: 500 mg/12-24 h) in monotherapy, both as recommended by Spanish guidelines [18], and beta-lactams + fluoroquinolone. Patients who received any other combination of antibiotics were classified as “other regimens”.

### Biological samples collection

In both the NCAP and non-infection control group, BAL fluid and blood samples were obtained on the day of bronchoscopic examination. In the CAP control group, blood samples were obtained at clinical stability, after at least 72 hours of antibiotic treatment, and no BAL fluid was obtained.

#### Bronchoalveolar lavage

BAL was collected according to recommended guidelines [19] by flexible videobronchoscope in the affected lobe in NCAP patients and in the middle lobe in non-infection controls. Five aliquots of sterile saline solution were instilled and immediately aspirated. The first aliquot (20 mL) was discarded. The remaining four aliquots (30 mL each) were pooled together in a single sterile glass: 50% of the retrieved fluid was sent to the microbiology laboratory and the other 50% to the biochemistry laboratory for cytokine measurement. The mean ± SEM BAL fluid obtained for processing was 56 ± 2 ml. To eliminate remaining mucus, the samples were filtered through two sheets of gauze. BAL fluid sample was centrifuged at 353 × g for 5 min at 4°C. The supernatant volume was measured and frozen at −80°C until further analysis.

#### Blood samples

Venous blood samples were collected prior to the BAL procedure and were centrifuged at 1500 rpm for 10 minutes. Plasma was separated, coded and frozen at −80°C until further processing.

### Cytokines determinations

The determination of cytokines levels [interleukin (IL)-6, IL-8, IL-10 and tumour necrosis factor α (TNF-α)] were analysed using the commercially available enzyme immunoassay (Pharmingen, BD Biociencias, Madrid, Spain) according to the manufacturer’s protocol. The limits of detection were 3 pg/ml for IL-6, 3 pg/ml for IL-8, 2 pg/ml for IL-10 and 1 pg/ml for TNF-α.

### Statistical analysis

Statistical analysis was performed with the SPSS statistical software package, version 12.0 (SPSS, Chicago, IL, USA), and the GraphPad software (San Diego, CA, USA). Chi-square and Mann–Whitney U tests were performed for, respectively, the qualitative and quantitative variables. Data are shown as mean ± SEM. Statistical significance was considered at a p value of <0.05.

## Results

### Patient characteristics

86 patients were included: 52 NCAP, 15 CAP controls and 19 non-infection controls. The main demographic characteristics, comorbidity, initial pneumonia severity and antimicrobial therapy for each group are shown in Table 1. The median days of hospitalization prior to enrolment in the study was 5 days in the NCAP group and 4 in the CAP control group. The reasons for non-response in the NCAP group were: 32 (61.5%) persistent fever and clinical worsening; 15 (28.8%) radiological progression- 2 of these with pleural effusion-; and 5 (9.6%) respiratory failure requiring invasive ventilation. Six patients died during hospitalization.

**Table 1:** **General characteristics, comorbidity, initial pneumonia severity and antimicrobial therapy of the study population**

	***NCAP***	***CAP control***	***Non-infection control***	p value*
Subjects, n	52	15	19	
Age, years	61 ± 2	66 ± 4	61 ± 2	NS
Male/Female	31/21	12/3	16/3	NS
Current smoker, n	16	2	9	NS
Comorbidity, n				
- Cerebrovascular disease	11(21)	3(20)	2(10)	NS
- Heart disease	12(23)	3(20)	2(10)	NS
- COPD	7(14)	7(46)	7(37)	0.01
PSI score	98 ± 4	102 ± 7	N/A	NS
CRP (mg/dl)			N/A	
- At admission	232 ± 25	190 ± 30		NS
- At enrolment	165 ± 18	68 ± 21		0.05
Inhaled corticosteroids	2(29)	1(14)	0(15)	NS
Systemic corticosteroids	2(29)	3(43)	0	NS
Antimicrobial therapy^β^, n			N/A	
- Fluoroquinolone	6(12)	5(33)		
- β-lactam plus macrolide	23(44)	9(60)		
- β-lactam plus fluoroquinolone	8(15)	0		
- Others regimens**	15(28)	1(7)		

Data are expressed as mean ± SEM or n(%). *Mann–Whitney *U* test and chi-square test for continuous and categorical variables, respectively.

NCAP: non-responders pneumonia; CAP: community-acquired pneumonia; COPD: chronic obstructive pulmonary disease; PSI score: Pneumonia Severity Index score; CRP: C-reactive protein; NS: non-significant. N/A: not applicable.

^β^Antimicrobial therapy at enrolment of the study.

Other regimens ^**^vancomycin, imipemen-cilastatin, piperacillin-tazobactam, amikacin, clindamycin, ceftazidime, ertapenem and linezolid.

General characteristics, comorbidity conditions and corticosteroids treatment according to macrolides regimens in the NCAP and CAP control groups are shown in Table 2. The median macrolide dosages for patients at the point of enrolment for the study were: 5 dosages of azithromycin 500 mg/24 h iv for the NCAP group and 3 dosages of azithromycin 500 mg/24 h iv for the CAP control group.

**Table 2:** **General characteristics, comorbidity and corticosteroids treatment in NCAP and CAP control groups according to macrolide containing regimens and non-macrolide regimens**

	***NCAP***			***CAP controls***		
	***Macrolide regimens*** ^***α***^	***Non-macrolide regimen*** ^***β***^	p value*	***Macrolide regimens*** ^***α***^	***Non-macrolide regimens*** ^***β***^	p value*
Subjects, n	23	29		9	6	
Age, years	60 ± 4	63 ± 3	NS	78 ± 3	70 ± 9	NS
Male/Female	12/11	19/10	NS	7/2	5/1	NS
Co-morbidity, n						
- Cerebrovascular disease	4(17)	7(24)	NS	3(33)	0	NS
- Heart disease	4(17)	8(28)	NS	2(22)	1(17)	NS
- COPD	1(4)	6(21)	NS	3(33)	4(67)	NS
Risk class of Fine			NS			NS
I-II	7(30)	6(21)		0	1(17)	
III-V	16(70)	23(79)		9(100)	5(83)	
Inhaled corticosteroid	0	2(7)	NS	1(11)	0	NS
Systemic corticosteroid	3(13)	6(21)	NS	4(44)	2(33)	NS

Data are expressed as mean ± SEM or n(%). *Mann–Whitney *U* test and chi-square test for continuous and categorical variables, respectively.

^α^Macrolide regimens: beta-lactam (ceftriaxone, cefotaxime or co-amoxi-clavulanate + macrolide (azithromicyn: 500 mg/24 h).

^β^Non-macrolide regimens: fluoroquinolone (levofloxacin: 500 mg/12-24 h) in monotherapy or beta-lactam + fluoroquinolone or other regimens.

NCAP: non-responders pneumonia; CAP: community-acquired pneumonia; COPD: chronic obstructive pulmonary disease; NS: non-significant. N/A: not applicable.

### Microbiological results

In the NCAP group, etiological microorganism was identified in 24 patients (46%). The most frequent isolated pathogen was *Streptococcus pneumoniae* (13 patients 25%): 3 in both blood culture and urinary antigen, 7 urinary antigen, 1 blood culture and 2 in BAL. The aetiological diagnosis with regard to macrolide regimens or otherwise, is provided in Table 3. In the CAP control group, aetiological diagnosis was reached in 5 patients (33.3%): 4 *Streptococcus pneumoniae,* 1 *Pseudomonas aeruginosa*.

**Table 3:** **Aetiological diagnosis in NCAP and CAP control group patients according to macrolide containing regimens and non-macrolide regimens**

	***NCAP***		***CAP control***	
	***Macrolide regimens***	***Non-macrolide regimens***	***Macrolide regimens***	***Non-macrolideregimens***
	***(n 23)***	***(n 29)***	***(n 9)***	***(n 6)***
Microorganisms				
- *Streptococcus pneumoniae*	5 (21.7)	8(27.6)	2(22.2)	2(33.3)
- *Pseudomonas aeruginosa*	2(8.7)	5(17.2)	1(11.1)	0
- *Escherichia coli*	0	2(6.9)	0	0
- *Streptococcus spp*	1(4.3)	1(3.4)	0	0
- MRSA	0	2(6.9)	0	0
- Polymicrobial	0	7(24.1)	0	0
Pathogens isolated	8(34.8)	16(55.2)	3(33.3)	2(33.3)

Date are presented as n (%).

NCAP: non-responders pneumonia; CAP: community-acquired pneumonia; MRSA: methilcilin-resistant *Staphylococcus aureus.*

### Cytokine levels in BAL and blood samples

#### NCAP group

**Figure 1:**
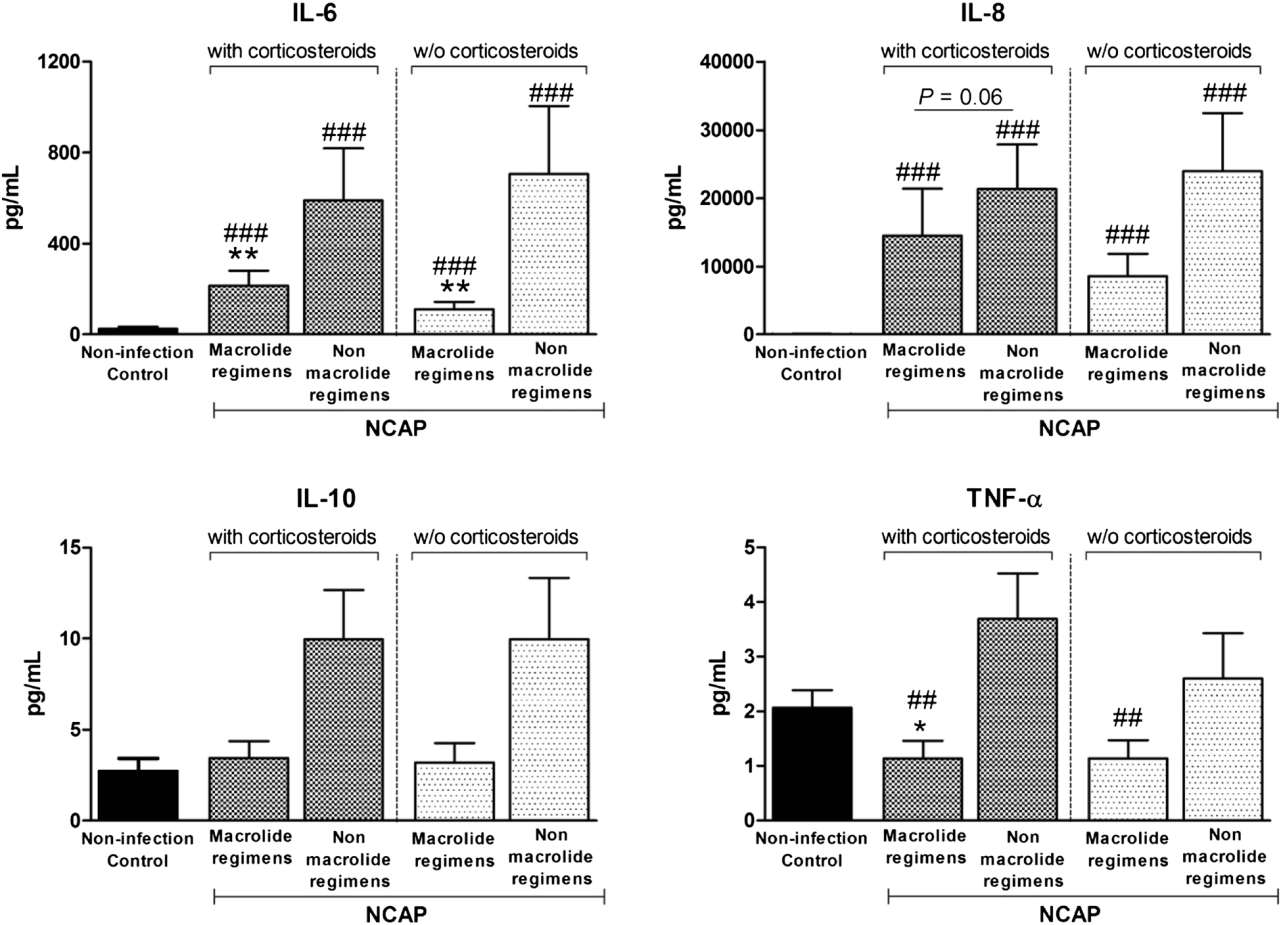
**BAL cytokine levels in the NCAP group according to macrolide containing regimens and non-macrolide regimens.** Data excluding corticosteroids users are also depicted. *Legend:* Data are presented as mean ± SEM. **/* macrolide regimens vs non-macrolide regimens; ###/## vs control. *: p < 0.05; **: p ≤ 0.01; ##: p < 0.05; ###: p < 001. NCAP: non-responders pneumonia; IL: interleukin; TNF-α: tumour necrosis factor-α.

The comparison between patients treated with and without macrolide regimens showed: In BAL fluid, IL-6 and TNF-α were significantly lower in patients treated with macrolide regimens (216 ± 66 vs 590 ± 230 pg/mL; p = 0.01 and 1 ± 0.3 vs 4 ± 0.8; p = 0.03 respectively), (Figure 1), with a trend towards lower IL-8 levels (p = 0.06)**.** After excluding patients with inhaled or systemic concomitant corticosteroids treatment, lower IL-6 levels in BAL (111 ± 32 vs 706 ± 300; p = 0.004) were confirmed.

**Figure 2:**
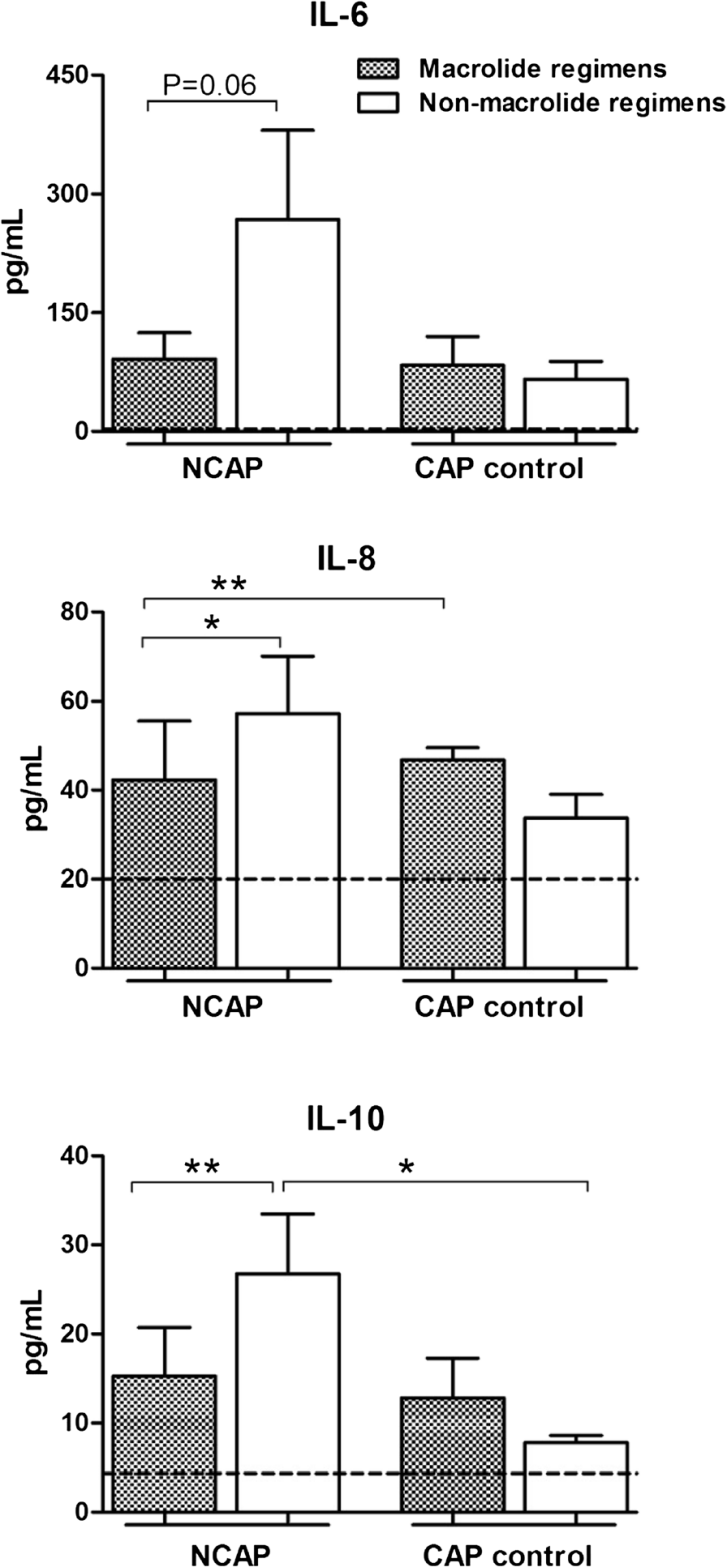
**Blood cytokine levels in NCAP and CAP control group according to macrolide containing regimens and non-macrolide regimens.** ***Legend:*** Data are presented as mean ± SEM. *: p < 0.05; **: p ≤ 0.01. NCAP: non-responders pneumonia; CAP: community-acquired pneumonia; IL: interleukin. Non-infection control group is represented as dashed line.

In blood, IL-8 and IL-10 were significantly lower in patients with macrolide regimens (42 ± 13 vs 57 ± 13; p = 0.04, and 15 ± 6 vs 27 ± 7; p = 0.01 respectively), (Figure 2), with a trend towards lower IL-6 levels (p = 0.06). No detectable levels of TNF-α in blood were obtained. In the subset of patients without corticosteroids concomitant treatment we corroborated lower IL-6, IL-8 and IL-10 levels in patients with macrolide regimens (96 ± 39 vs 305 ± 137, p = 0.05; 35 ± 14 vs 52 ± 14, p = 0.05; and 14 ± 6 vs 23 ± 6, p = 0.02, respectively).

#### CAP control group

No significant differences in blood cytokine levels were observed between patients treated with macrolide containing regimens and those treated with non-macrolide regimens (Figure 2) in the whole group and after excluding patients with corticosteroid concomitant treatment. Similarly, no differences were observed between COPD and non-COPD patients.

### Clinical outcomes

The relationship between the days needed to reach clinical stability and LOS according to treatment with macrolide and non-macrolide regimens in the NCAP group and the CAP control group is shown in Table 4.

**Table 4:** **Comparison of clinical outcomes in NCAP and CAP control group according to macrolide containing regimens and non-macrolide regimens**

	***Clinical stability (days)***	***p#***	***LOS***	***p#***
***NCAP***				
-Macrolide regimens *(n 23)*	8(6–12)	0.007	12(10–21)	0.007
-Non-macrolide regimens *(n 29)*	14(8–27)		20(13–36)	
***CAP control***				
-Macrolide regimens *(n 9)*	6(3–10)	0.6	9(7–16)	0.8
-Non-macrolide regimens *(n 6)*	6(3–6)		9(8–12)	

Data are presented as median and interquartile range. *p*# values were calculated using the Mann–Whitney *U*-test.

NCAP: non-responders pneumonia; CAP: community-acquired pneumonia; LOS: Length of stay.

In the NCAP group, patients with macrolide containing regimens required fewer days than patients with non-macrolide regimens to reach clinical stability, and had shorter LOS. After excluding patients with corticosteroid concomitant treatment we confirmed significantly shorter LOS in patients treated with macrolide regimens (13 ± 1.5 vs 24 ± 4, p = 0.01, respectively) and fewer days to reach clinical stability (8 ± 1 vs 16 ± 3, p = 0.004, respectively). No differences were found in the whole CAP control group and after excluding those with corticosteroid treatments.

## Discussion

The most important findings of our study are: 1) Levels of IL-6 and TNF-α in BAL and of IL-8 and IL-10 in blood were significantly lower in NCAP patients who received macrolide containing regimens than in those treated with non-macrolide regimens; and 2) there were improved clinical outcomes, such as earlier clinical stability and shorter LOS, in patients who received macrolide containing regimens. Our main findings were also confirmed in patients without any concomitant corticosteroid treatment.

In this study, patients who did not reach clinical stability at 72 hours after antibiotic treatment developed raised cytokine inflammatory levels in both compartments, pulmonary and systemic, thereby following a pattern similar to that found in previous reports. Persistently high levels of IL-6, IL-8 and IL-10 in BAL fluid or serum have been found in severe pneumonia and NCAP [2,4,20,21], reflecting an ongoing inflammation. In this context, declining levels of biomarkers, IL-6, IL-8 and IL-10, along with apoptosis of neutrophils have also been reported at clinical stability [22].

The beneficial impact of controlling inflammatory response through immunodulatory agents in CAP has received significant attention [5,23-26]. In our study, NCAP patients treated with macrolide containing regimens exhibited a trend towards IL-8 BAL fluid levels and significantly lower IL-6 and TNF-α compared to patients treated with non-macrolide regimens. Moreover, in blood our results also confirmed lower levels of IL-6, IL-8 and IL-10 in the NCAP patients treated with macrolide regimens, while in those CAP control patients who attained clinical stability no significant differences were detected.

Despite the recognized role of macrolides in tempering inflammation, there are very few studies on humans of this [25]. In cases of CAP, Demartini *et al.* [27] compared systemic cytokines levels of clarithromycin (500 mg twice a day for 7 days) and amoxicylin (1 g three times a day for 7 days) in patients before starting antibiotic therapy, at the 3rd and 7th days of therapy. Clarithromycin decreased significantly levels of IL-6 and increased levels of IL-10 at the 3rd and 7th days in comparison with basal levels. In a randomized clinical study of ventilator associated pneumonia patients, Spyridaki *et al.* [28] analyzed the effect of the clarithromycin and placebo on cytokines over six consecutive days. The serum ratio of IL-10 to TNF-α decreased in the clarithromycin group compared to the placebo group.

It is also worth highlighting that in our study the effect of macrolides was not apparent in CAP patients with clinical stability, which indicates that, at that point, cytokine levels were already reduced.

Macrolides inhibit intracellular signaling pathways, suppress the production of NF-kB and the synthesis and/or secretion of pro-inflammatory cytokines, and decrease influx and neutrophil activity [23]. In animal models with pneumonia, the reduction of chemokine secretion and cytokine levels was demonstrated when microorganisms were resistant to macrolides, suggesting that benefits were independent of antimicrobial activity [29]. Sanz *et al.* [30], reported in respect of an animal study *in vivo* that erythromycin exerts anti-inflammatory activity and inhibits leukocyte recruitment in the lung. In *in vitro* studies, results suggest that macrolides exhibit a suppressive effect on cytokines in models of acute inflammation [25], viral bronchiolitis [31] and ventilator-induced lung injury [32]. Anderson *et al.* [33], have reported that the production of pneumolysin, a key virulence factor of the pneumococcus, is attenuated by exposure of this microbial pathogen to clarithromycin.

The beneficial impact of macrolides on outcomes in CAP cases has been recently evaluated in a systematic review [23] that identified 6 uncontrolled studies: 4 in favor of macrolides [12,34-36] and 2 without beneficial effects [13,14]. Moreover, in severe sepsis due to CAP, Restrepo *et al.* [10], found that patients who received macrolide therapy in comparison with those patients who did not receive it were associated with decreased mortality at 30 days (11% vs 29%, p < 0.001) and at 60 days (12% vs 34%, p < 0.001) even in those cases with macrolide-resistant pathogens. Recently, Rodrigo *et al.* [37], studied the benefits of beta-lactams/macrolide combination therapy over single beta-lactams therapy for the treatment of immunocompetent adults hospitalized with CAP. They reported that the 30 days in-patient death rate was lower in the combination therapy than in the single therapy group (23% vs 26.8%; OR 0.81, 95% CI 0.72 to 0.93, p = 0.001). In bacteremic pneumonia Metersky *et al.* [11], reported that the use of macrolides was associated with lower in-hospital mortality, 30-day mortality and readmission within 30 days of discharge.

Interestingly, the gain in survival and improved outcomes with regard to dual antibiotic therapy, mainly in severe and bacteremic pneumococcal CAP, is associated principally with treatments that include macrolides [38] instead of beta-lactams plus fluoroquinolones [39]. This is probably due to the immunomodulatory effect of macrolides.

*In vitro* and *in vivo* studies have shown that, due to their immunomodulatory effects, macrolides decrease inflammatory response, independently of antibacterial activity, through different pathways: inhibition of NF-kβ along with reduction of proinflammatory cytokines production; an inhibitory effect on the release of inflammatory cells such as polymorphonuclear cells; and an effect on structural cells of the respiratory tract that improves mucociliary clearance and increases the expression of molecules tight junctions or β-defensin [24]. In addition, macrolide antibiotics have an effect on microorganisms because they may inhibit virulence factors production, biofilm formation and protein synthesis [24]. Moreover, a macrolide combination therapy could provide better coverage for atypical microorganisms.

Some other important factors can influence the inflammatory response in CAP patients, such as corticosteroids treatment and comorbidity as COPD [40]. In our study, we corroborated our results with lower IL-6 in BAL and IL-8 and IL-10 in blood after excluding patients treated with corticosteroids.

Our findings are consistent with previous studies as they show an improvement of clinical outcomes (faster clinical stability and shorter LOS) and a reduction of cytokine levels in CAP patients with acute lung infection who are treated with macrolide containing regimens. These findings could be related to decreased levels of the pro-inflammatory cytokines IL-6, IL-8 and TNF-α, and support the value of macrolides for their beneficial effect over immunomodulatory properties.

The present study has some limitations that should be considered.

First, the absence of BAL fluid samples in the CAP control group as, for ethical reasons, we did not perform a bronchoscopy in patients with adequate response. There are few human studies that report compartmental inflammatory response in patients with CAP, probably because of the ethical difficulties of performing BAL in non-severe CAP. The current guidelines for management of CAP do not recommend invasive studies when clinical response is appropriate. Second, for our study we enrolled patients after they had received antibiotic treatment. We therefore did not have cytokine levels for those patients prior to initiating their treatment. Third, to improve the robustness of our results, we could ideally have been controlled for patient age and pneumonia severity. However, our study draws upon a single-hospital cohort, and hence upon a limited number of patients. This, and the aforementioned difficulties in collecting data from NCAP patients, including the BAL procedure, prevented our being able to conduct a multivariate analysis for this study.

## Conclusions

Our study shows that after 72 hours of antibiotic therapy, NCAP patients treated with macrolide containing regimens have lower cytokine levels in both compartments (systemic and pulmonary) than those treated with non-macrolide regimens. This supports the immunomodulatory effect of macrolides on cytokine profiles during the course of treatment. That effect seems to contribute to faster resolution and earlier clinical stability. Further randomized trials are needed to confirm the benefit of macrolide therapy in NCAP patients.

## Abbreviations

BAL: Bronchoalveolar lavage
CAP: Community-acquired pneumonia
COPD: Chronic obstructive pulmonary disease
IL: Interleukin
LOS: Length of hospital stay
NCAP: Non-responders CAP
TNF-α: Tumour necrosis factor-α
